# Exhaled Breath Analysis in Diagnosis of Malignant Pleural Mesothelioma: Systematic Review

**DOI:** 10.3390/ijerph17031110

**Published:** 2020-02-10

**Authors:** Zehra Nur Töreyin, Manosij Ghosh, Özlem Göksel, Tuncay Göksel, Lode Godderis

**Affiliations:** 1 University of Leuven (KU Leuven), Department of Public Health and Primary Care, Centre for Environment and Health, 3000 Leuven, Belgium; manosij.ghosh@kuleuven.be (M.G.); lode.godderis@kuleuven.be (L.G.); 2 Ege University, Faculty of Medicine, Department of Pulmonary Medicine, Division of Immunology, Allergy and Asthma, Laboratory of Occupational and Environmental Respiratory Diseases, Bornova, 35100 Izmir, Turkey; goksel.ozlem@gmail.com; 3 Ege University, Faculty of Medicine, Department of Pulmonary Medicine, Bornova, 35100 Izmir, Turkey; tuncaygoksel@gmail.com; 4 Idewe, External Service for Prevention and Protection at Work, 3001 Heverlee, Belgium

**Keywords:** malignant pleural mesothelioma, exhaled breath analysis, volatile organic compounds, exhaled breath condensate

## Abstract

Malignant pleural mesothelioma (MPM) is mainly related to previous asbestos exposure. There is still dearth of information on non-invasive biomarkers to detect MPM at early stages. Human studies on exhaled breath biomarkers of cancer and asbestos-related diseases show encouraging results. The aim of this systematic review was to provide an overview on the current knowledge about exhaled breath analysis in MPM diagnosis. A systematic review was conducted on MEDLINE (PubMed), EMBASE and Web of Science databases to identify relevant studies. Quality assessment was done by the Newcastle–Ottawa Scale. Six studies were identified, all of which showed fair quality and explored volatile organic compounds (VOC) based breath profile using Gas Chromatography Coupled to Mass Spectrometry (GC–MS), Ion Mobility Spectrometry Coupled to Multi-capillary Columns (IMS–MCC) or pattern-recognition technologies. Sample sizes varied between 39 and 330. Some compounds (i.e, cyclohexane, P3, P5, P50, P71, diethyl ether, limonene, nonanal, VOC IK 1287) that can be indicative of MPM development in asbestos exposed population were identified with high diagnostic accuracy rates. E-nose studies reported breathprints being able to distinguish MPM from asbestos exposed individuals with high sensitivity and a negative predictive value. Small sample sizes and methodological diversities among studies limit the translation of results into clinical practice. More prospective studies with standardized methodologies should be conducted on larger populations.

## 1. Introduction

Malignant pleural mesothelioma (MPM) is a rare but aggressive tumor arising from mesothelial cells of pleural membranes [1]. Globally, 30,443 new MPM cases were estimated from the GLOBOCAN database in 2018 [2]. Mortality trajectories show an increase in annual rates of MPM deaths globally, contrary to popular belief [3,4]. It has been more than 50 years since Wagner et al., established the relationship between asbestos fibers and histologically proven MPM in the miners and their households who employed in asbestos rich areas of South Africa [5]. Doll et al., have linked the lung cancer diagnosis of a group of workers with their past asbestos exposure [6]. It has been well established that exposure to mineral fibers such as asbestos and erionite is the main cause of MPM [7]. Asbestos is the commercial umbrella term for six types of naturally occurring silicate fibers, all of which have been considered as carcinogenic to humans by International Agency for Research on Cancer (IARC) [8]. Erionite is another naturally occurring mineral fiber belonging to the zeolite minerals. Its chain-like structure resembles that of amphibole asbestos and was found to have more potent carcinogenic effects on human mesothelial cells [9,10]. Indeed, all types of mineral fibers showing asbestiform disposition have potential to cause lung cancer and MPM, depending on their aspect ratio (length to width) and bio-persistence [11,12]. 

**Figure 1:**
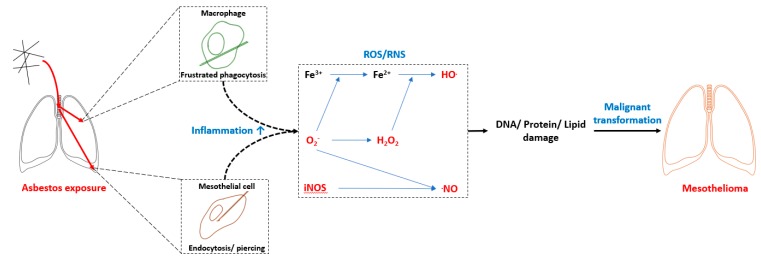
Asbestos induced inflammatory process. ROS—reactive oxygen species; RNS—reactive nitrogen species; iNOS—inducible nitric oxide synthase.

Asbestos fibers migrate towards pleura following inhalational exposure and may incite carcinogenesis independent of a safe exposure limit. Several hypotheses have been proposed to explain asbestos-induced diseases. The most accepted ones include the formation of highly reactive hydroxyl radical through the Haber–Weiss reaction which is produced by the interaction of superoxide (O_2_^−^) and hydrogen peroxide (H_2_O_2_) and catalyzed by asbestos surface iron. In addition, reactive oxygen radicals (ROS), generated mainly by macrophages, but also by lung fibroblasts and mesothelial cells, contribute to asbestos-induced inflammation [13,14]. Persistent accumulation of ROS may cause abnormal DNA repair and DNA damage, leading to carcinogenesis (Figure 1). Moreover, Tumor Necrosis Factor TNF-α may activate nuclear transcription factor (NF-κB) which increases the durability of mesothelioma cells and hence, eases their duplication. Asbestos also promotes the phosphorylation of mitogen-activated protein kinase and extracellular signal-regulated kinases 1 and 2, generating the over expression of proto oncogenes. In addition, research on mesothelioma genome supports the role of inactivated tumor suppressor genes encoded by cyclin-dependent kinase inhibitor 2A (CDKN2A), BRCA1 associated protein 1 (BAP1) and neurofibromin 2 (NF2) [15,16,17].

Patients with asbestos exposure history along with compatible computerized tomography (CT) findings for mesothelioma usually undergo thoracoscopy for histological investigation and tumor staging. However, due to morphological heterogeneity of tumor cells, even sufficient tissue may remain inadequate for the exact diagnosis. In addition, metastasis from other sites or reactive effusions may complicate diagnosis [18]. Therefore, it is difficult to detect MPM at the very early stages. In addition to the histological challenges, thoracoscopy can be contraindicated in a group of patients. For instance, owing to the insidious course of MPM, patients are generally admitted in advanced or at terminal stages, with poor performance scores ruling out an invasive procedure. In addition, MPM is prominent in the elderly who likely have multiple comorbidities. 

Immune cytochemical markers in tissue or effusion are recommended to distinguish benign versus malignant mesothelial proliferations and mesothelioma versus other malignancies. At least two positive markers for mesothelioma (i.e., calretinin, cytokeratin 5/6, Wilms Tumor (WT1) and podoplanin (D240) and at least two negative markers for adenocarcinoma (thyroid transcription factor-1, carcinoembryonic antigen and Ber-EP4 antibody) are recommended as complimentary to other diagnostic procedures [18].

Due to the challenges with diagnosis and unfavorable effect of late diagnosis on disease outcome, as well as the predicted burden of MPM in the future, there is excessive research interest in the discovery of potential biomarkers of MPM. Among them, studies on tissue and blood biomarkers (i.e., mesothelin, osteopontin, fibulin-3, high mobility group box-1 (HMGB1) protein, aquaporin 1, microRNAs, proteomics-based biomarkers) have shown promising results [19,20,21,22,23,24,25,26]. Soluble mesothelin related peptide (SMRP) is the most studied biomarker and has been approved by the Food and Drug Administration (FDA) to be used in MPM diagnosis. It is expressed as membrane-bound peptides from healthy mesothelial cells and becomes detectable in the bloodstream after cleavage of the membrane-bound mesothelin. Although the role of mesothelin within mesothelioma carcinogenesis is ambiguous, several hypotheses emphasize its role within invasion via interaction with mucin MUC16 and the NF-κB signalling pathway [13]. Some studies on the diagnostic value of SMRP in asbestos exposed population reported a 68.2–75% sensitivity and 80.5–96.2% specificity in distinguishing MPM with benign pleural diseases [19,20,21]. Renal failure was found to affect its serum level [19]. Moreover, particular polymorphisms within the MSLN gene were found to be related with high levels in healthy subjects [22,23]. Hence, clinical findings should be addressed carefully when interpreting high serum levels. The duration of asbestos exposure was weakly or not correlated with SMRP levels in several studies [20,24]. In a meta-analysis, the overall sensitivity and specificity of osteopontin in MPM diagnosis were found 0.65 (95% CI: 0.60–0.70) and 0.81 (95% CI: 0.78–0.85), respectively [27]. Some recent studies have shown that the combined use of these potential biomarkers in asbestos exposed population may improve the accuracy of the diagnostic performance of individual markers [28,29,30,31].

Despite these efforts, there is still dearth of information on non-invasive biomarkers to detect early metabolic and inflammatory changes of MPM in asbestos-exposed individuals. In the context of MPM management, a good biomarker should be sensitive and specific enough to discriminate an MPM patient from a healthy person. Moreover, it should be predictive enough to catch the early precursors of malignant transformation in an asbestos-exposed population. The latter would avoid a group of asbestos exposed workers unnecessarily undergoing invasive diagnostic procedures. 

The aim of this review is to provide an overview on the current knowledge about the use of exhaled breath analysis in MPM diagnosis as well as to give insights into potential histopathologic backgrounds of the breath profile changes detected. Hence, in the following sections, we will summarize the methodological aspects of exhaled breath analysis and the findings of available studies.

## 2. Exhaled Breath Analysis 

### 2.1. Exhaled Breath Composition 

**Figure 2:**
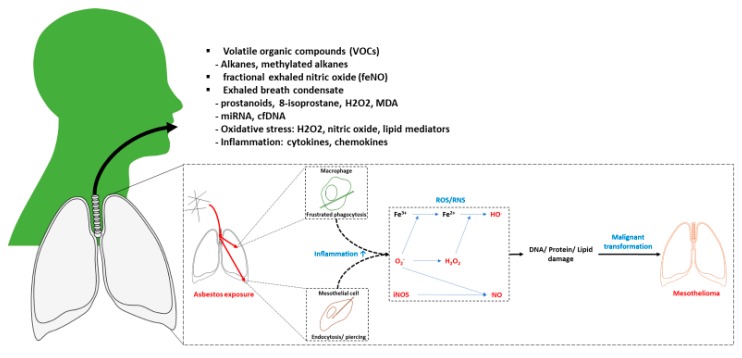
Exhaled breath composition. MDA—malondialdehyde.

Exhaled breath is the biological matrix for thousands of markers of exogenous or endogenous origin. In its physical structure, it is composed of three compartments: volatile fraction, gaseous fraction (i.e., fractional exhaled breath—FeNO) and exhaled breath condensate (EBC) [32]. Each of these components carries different biochemical products reflecting lung and peripheral body metabolism. For instance, EBC reflects airway lining biofluid composition while volatile fraction carries numerous organic compounds which have high vapor pressures and become volatile in ambient temperatures [33]. Different sampling and pre-treatment methods are implemented when studying these fractions (Figure 2). 

Volatile organic compounds (VOCs) constitute the volatile fraction found in exhaled breath in very small amounts ranging from parts per trillion volume (pptv) to parts per billion volume (ppbv) [32,34]. They can be generated through metabolic processes of biologic systems, environmental sources (i.e., fuel combustion, cigarette smoke, fragrances, ingestion of drinks or food, drugs) or the metabolism of airway and gut microbiome. VOCs generated through metabolic pathways may arise from the respiratory system or from other systemic sources [35]. Compounds of the systemic origins dissolve into the blood and transport from alveolar circulation into alveolar space by a passive diffusion mechanism. The relative concentrations in alveolar space in blood and in adipose tissue are determined by their “fat-to-blood” and “blood-to-air” partition coefficients. The latter also indicates whether organic compounds in blood can be excreted via breath or not [34,36]. In addition, cardiac output and alveolar minute volume of subjects also influence VOC concentrations in exhaled breath. Exposure to several environmental contaminants can also be detected based on identification of the substance or metabolites in the breath (i.e., measurement of exhaled methyl-tertiary butyl ether (MTBE) and its metabolite tertiary butyl alcohol (TBA) in the exhaled breath of gasoline workers) [37].

The identification of VOCs related with tumor metabolism show a promising future in the screening and diagnosis of several cancers [34]. Possible mechanisms of cancer-related VOC emissions include (1) persistent oxidative stress-induced lipid peroxidation of polyunsaturated fatty acids (PUFAs) (i.e., emission of alkanes, alkenes and aldehydes following cell- and mitochondrial-membrane perturbations by ROS attacks) (2) induction of cytochrome p-450 enzymes by environmental carcinogens and ROS molecules (3) overexpression of cytochrome p-450 enzymes in several tumor types, such as breast tumors, (4) cancer cell metabolism shift from oxidative phosphorylation to aerobic glycolysis, (5) molecular alterations in oncogenes and tumor suppressor genes [34,38,39,40,41]. 

VOCs have been extensively studied in lung cancer in vivo and in vitro. Of these identified compounds, many studies reported lipid peroxidation (i.e., decane, heptanal, octane, undecane) and mevalonate (i.e., isoprene) pathways, related ones being high in lung cancer compared to healthy controls [41,42,43]. Moreover, it has been suggested that [41] peroxidation processes may produce cancer-specific VOCs given the hypothesis that lung cancer cells present altered phospholipid profiles compared to that of healthy cells [44]. However, none of these markers have been translated into clinical practice yet. 

Fractional exhaled nitric oxide (FeNO) reflects the inflammatory condition of airways. A few studies have shown elevated FeNO levels in asbestos-exposed individuals [45,46,47].

EBC is another breath-derived matrix collected by cooling exhaled air at temperatures of 4 °C or lower, during spontaneous breathing [33,48]. Its main constituent is water, which makes EBC a highly diluted biofluid, while the remaining part is constituted by respiratory droplets carrying oxidative stress-related biomarkers (H_2_O_2_, 8-isoprostane), proinflammatory and inflammatory mediators (NOx, prostanoids, leukotriens), DNA and miRNA. EBC composition was proposed to be reflective of the airway lining fluid making up the essential part of the pulmonary host defense system [33,48,49]. EBC analysis is also promising in occupational settings as it provides non-invasive and repeatable access to biomarkers of exposure and effect [50]. Its constituents and volume are supposed to be affected by circadian rhythm, age, sex, diet and drugs as well as pulmonary and extra-pulmonary diseases; hence, studies with human samples should account for these potential confounders [33].

Oxidative stress and inflammatory biomarkers, particularly 8-isoprostane and H_2_O_2_, were reported to be consistently high in the EBC of former asbestos-exposed individuals in several studies [15,45,47]. Of these, 8-isoprostane is generated following perturbation of membrane arachidonic acids by free radicals and is hence presumed to be more specific to lipid peroxidation compared to other oxidative stress biomarkers [48,51]. In addition, higher LTB4 levels were found in the EBC of asbestos-exposed individuals. One study reported a correlation between EBC LTB4 levels and the severity of radiological findings and lung function impairment in asbestosis patients [52].

Human studies on the exhaled breath biomarkers of cancer and asbestos-related diseases have shown encouraging results. However, limitations caused by unstandardized methodologies and small population sizes of the studies are likely to hamper their translation into clinical practice.

### 2.2. Sample Collection

A breath sample can be collected by polymeric bags, canisters or sorbent tubes to analyze volatile fraction [53]. Polymeric bags are made up of inert materials in order to avoid reactions with the breath sample and have the advantage of a low cost and ease of use. However, contamination with phenol, N,N-dimethylacetamide, carbon disulfide, and carbonyl sulfide was reported with some types [53,54]. The use of disposable bags would reduce contamination with cleaning agents. Moreover, the use of inert gases (i.e., ultra-pure nitrogen) for cleaning has been shown to be effective for repeated uses of polymeric bags [55]. Canisters are durable and avoid degradation of breath. However, they are expensive and need specific agents for cleaning, that might emit VOCs [53,55]. 

Breath can be sampled directly onto an analytical hardware or collected and concentrated through pre-treatment procedures before analysis [53,56]. Pre-concentration methods include VOC adsorption onto sorbent-containing thermal desorption (TD) traps or via solid-phase microextraction fibers (SPME) [53].

There are several issues to be considered during VOC sampling. The first is correction for exogenous volatiles, which can be in part achieved by subtracting ambient VOC concentrations from exhaled VOC concentrations or by breathing inhalation/exhalation filters before sampling. Decision on breath sample type is another issue to be considered as alveolar breath is more reflective of endogenous VOCs compared to that of total breath. Alveolar fraction can be obtained by disposing of the first 150 ml of forced-expiratory air samples corresponding to dead space [57] or ideally, by continuous monitoring of CO_2_, which is reflective of alveolar-gas exchange. The latter is based on targeted breath sampling in parallel with the end-tidal CO_2_ levels [32,58]. Moreover, some commercial breath sampling devices are able to capture the end-tidal air [32]. Sampling duration and breathing pattern should also be considered during sampling as they may affect the breath profile [53].

During the sampling process of EBC, subjects breathe tidally over a predetermined period of time into a condensate/cooler device coated with either teflon, polypropylene, glass, silicone or aluminium [55,57]. It is recommended to refrain from exercise at least one hour in advance in order to decrease breath flow rates. In addition, periodic swallowing is recommended to avoid saliva contamination. Breath can also be affected by volatile constituents within ambient air; hence, a filter applied onto inhalation valve can help to reduce the environmental contamination of EBC. It is suggested to analyze the condensate as immediately as possible. Otherwise, freeze-drying and storage of the samples at −80 °C applied right after sampling are recommended procedures [55]. If the samples have been at −80 °C until analysis, transferring the samples into capped glass tubes within a controlled environment (i.e., via a laminar flow cabinet) before storage is recommended in order to decrease possible sample contamination by environmental air) [59]. 

### 2.3. Detection Methods 

Exhaled breath detection can be addressed via two distinct methods. The first involves analytical techniques based on the identification of individual compounds. The second is pattern recognition of VOCs by utilizing sensor technology which—contrarily to analytical chemical methods—does not identify specific compounds [36,55]. 

#### 2.3.1. Analytical Methods 

Gas Chromatography Coupled to Mass Spectrometry (GC–MS) is still accepted as the golden standard method for VOC analysis and detects an extensive range of VOC profiles with high sensitivity and specificity. The operating principle is based on separation of VOCs by their chemical properties, subsequent ionization, fragmentation and identification of compounds according to their mass-to-charge ratio. The retention time, which is the time point to reach the end of GC column, differs among VOCs and allows the identification of individual compounds in a mass-spectra library. Disadvantages of GC–MS include its high cost and requiring expert technicians. In addition, GC–MS is not convenient for point-of-care use as it is not portable and do not provide real-time assessment [55,56]. Other analytical methods include selected ion flow-tube MS (SIFT–MS), proton-transfer reaction MS (PTR-MS) and ion-mobility spectrometry (IMS) which enable chemical ionization of breath molecules with H_3_O^+^, NO^+^, or O^2+^ under very well controlled reactions, hence eliminating the need for a chromatographic process and providing a real-time assessment [35,55,56]. Sometimes, they are coupled to multi-capillary columns (MCCs) that allows a separation of complex mixtures more rapidly than GC [35,60]. PTR-MS has the advantage of a higher sensitivity over SIFT–MS. However, both methods are less sensitive in a VOC analysis compared to GC–MS. 

#### 2.3.2. Pattern-Recognition-Based Methods 

E-nose has been utilized as an electronic detection system for breath analysis. It is composed of non-selective sensor arrays detecting VOCs. Sensors produce fingerprints of these compounds according to their chemical and electronic properties. By utilizing the “probabilistic workflow approach”, fingerprints are transformed into digital data and processed by several pattern recognition techniques in order to identify breath profiles pertinent to several diseases [58,61]. E-nose has the advantage of being portable and allowing real-time assessment of human breath composition; hence, is likely to have the potential to be translated into point-of-care use in clinical settings. Several studies have shown good sensitivity and specificity with e-nose in discriminating the breath profiles of respiratory diseases from those of healthy subjects [62,63,64]. The disadvantage of eNose is that it does not allow the identification of specific compounds that would have the role in pathophysiological pathways of diseases. On the other hand, it allows diagnosis and monitoring of diseases based on fingerprints being recognized. 

## 3. Methods 

### 3.1. Search Strategy

This systematic review was conducted in accordance with Preferred Reporting Items for Systematic Review and Meta-Analysis (PRISMA) guidelines [65]. Search concepts were determined according to the “PECO” strategy (Table 1). An electronic search was carried out using MEDLINE (PubMed), EMBASE and Web of Science databases. Papers published from 2000 to September 2019 were screened. The search strategy consisted of a combination of controlled search words (e.g., Medical Subject Headings/MeSH) and free-text words to specify three search strings: ‘mesothelioma’, ‘pleural mesothelioma’, ‘malignant pleural mesothelioma’ combined with ‘exhaled breath’, ‘breath tests’, ‘gas chromatography’, ‘mass spectrometry’, ‘exhaled breath condensate’, ‘breathomics’, ‘proteome’, ‘phosphoproteomics’, ‘proteomics’ (inception until September 2019). Search terms were modified and proper MeSH terms were selected with the help of a librarian in KU Leuven. In addition, the reference lists of selected articles were checked for any additional studies to include. A methodological filter was not applied. The complete search strategy is presented in Table S1 in the Supplementary Section.

### 3.2. Study Selection

The titles and abstracts of the studies that are pertinent to search terms were retrieved and screened by two independent authors (Z.N.T and M.G.) against eligibility criteria. Studies were included if they met the following criteria:

1. Studies in English
2. Studies on human samples
3. Studies that included pleura biopsy confirmed MPM patients
4. Studies that assessed diagnostic accuracy of exhaled breath methods in diagnosis and/or prognosis of MPM. Studies were excluded if a diagnostic marker discovery was performed only in biofluids other than exhaled breath.

Review articles, meta-analyses, case reports, case series, meeting reports and conference abstracts were excluded.

### 3.3. Data Extraction and Synthesis

The full texts of selected studies were reviewed by two independent authors (Z.N.T. and M.G.). Any discrepancies were resolved through discussion and if required, a third reviewer (L.G.) was involved. 

Descriptive data regarding: (1) Study details (date of study, title, author and research question) (2) Methods (study design, exposure, primary outcome, potential confounders and any other outcomes) (3) Patient population (population demographics, sample size, inclusion and exclusion criteria) (4) Exposure assessment method (5) Type of exhaled breath analysis (identification of VOC constituents via analytical methods, sensor-based pattern recognition technology, proteomics expressions via exhaled breath condensate) (6) Sensitivity, specificity, area under the receiver operating characteristics (ROC) curves of diagnostic approaches were extracted and recorded on excel sheets dedicated to each study. (7) Sensitivity and specificity rates were grouped according to VOC approaches and forest plots were generated using Review Manager version 5.3. (The Cochrane Collaboration, Copenhagen, Denmark) [66].

### 3.4. Quality Assessment 

The quality of articles regarding their observational study design was evaluated using the Newcastle–Ottawa Scale (NOS) [67]. NOS is a tool developed for the quality assessment of non-randomized studies. It has three main domains concerning the “selection” of the study groups, “comparability” and “exposure assessment” of cases and controls. Each domain includes numbered items and evaluation is based on the allocation of a star (*) to each item, indicating favorable judgement. We modified the comparability domain considering age, sex, smoking status and the presence of additional systemic conditions that would affect constituents of exhaled breath. In addition, we added exposure information from company records into the “ascertainment of exposure” item. Selection and exposure domains can be awarded with a maximum of four and three stars, respectively, while the comparability domain can be awarded with a maximum of two stars. An overall score of ≥7 defined high quality, 4–7 fair quality, and 0–3 poor quality.

## 4. Results 

**Figure 3:**
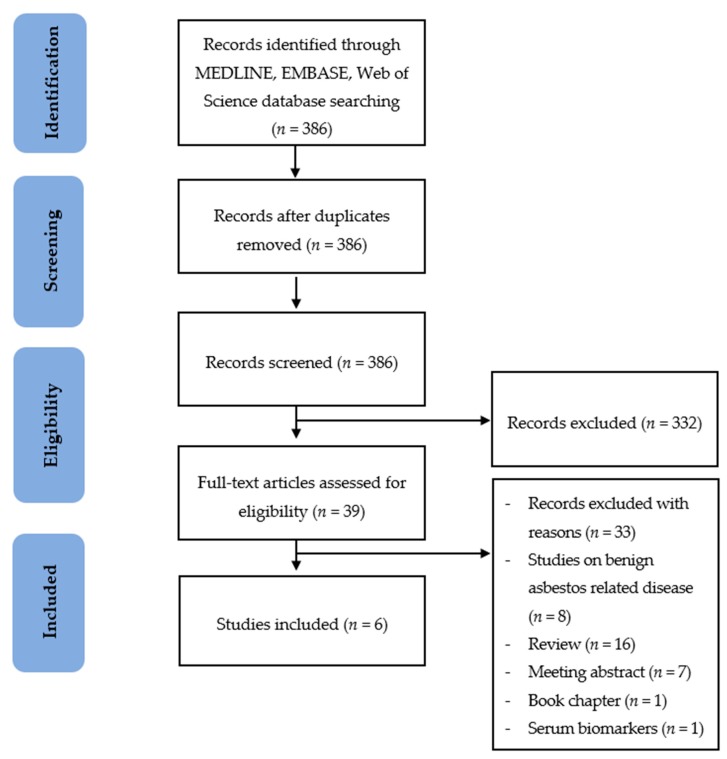
Flow chart of the study selection process.

In total, 386 records compatible with the search terms were identified through the MEDLINE, EMBASE and Web of Science databases. These records were subsequently exported to Endnote X9 (Philadelphia, PA, USA) [68]. Ten additional records were identified through reference lists of relevant articles. A total of 368 of them remained for title and abstract screening after duplicates were excluded. Studies that did not meet the inclusion criteria were excluded during title and abstract screening and thirty-nine were closely assessed for eligibility. Of these thirty-nine records, sixteen review articles, seven meeting abstracts and one book chapter were excluded. One study was excluded because it solely investigated serum biomarkers of MPM. The remaining eight studies did not cover MPM patients, although they assessed exhaled breath analysis of asbestos related diseases. Six studies [69,70,71,72,73,74] were eventually included in the review. An overview of the study selection steps is presented in Figure 3.

### 4.1. Quality Assessment of Studies

The overall quality scores of studies ranged from 4 to 6 (out of 9), indicating fair quality. Representative samples of MPM cases were recruited from respiratory and occupational medicine departments of several hospitals, in all the studies. Control groups were selected from volunteers working at hospitals [69,70], from the patients visiting outpatient settings [72,73,74] or from community volunteers [71]. Cases and controls were matched for age, sex and smoking status in one study [71]. Three studies excluded subjects with any systemic disease or respiratory infection [69,70,71]. Information regarding asbestos exposure of MPM cases were obtained through self-reports [69,70] or questionnaires [71,72,73,74] while that of asbestos exposed control groups were mainly retrieved from company records [69,70,72,73,74]. However, none of the studies obtained asbestos exposure information through structured exposure assessment tools nor accounted for duration of asbestos exposure. Moreover, none of the studies ensured blindness of the investigators. The results of the NOS scores of the included studies are presented in Table 2. In addition, the details of the quality assessment are presented in Table S2 in the Supplementary Section.

### 4.2. Study Characteristics 

All studies were of a cross-sectional, case-control design, published between 2010 and 2017, and explored VOC-based exhaled breath profile in MPM patients using analytical or sensor-based technologies. Four studies [71,72,73,74] were conducted at more than one center. The number of MPM cases varied between 13 and 52 across studies. All MPM diagnosis were confirmed by pleural biopsy. In addition, a group of pathology panel was asked for independent confirmation in three studies [72,73,74], which conformed to international guidelines [75]. Control groups composed of asbestos-exposed workers (AEx) and never asbestos-exposed healthy individuals (HC). Five studies [69,70,71,73,74] included subjects with benign asbestos-related diseases (ARD). In addition to AEx and ARD controls, the breath profiles of MPM patients were compared with those of lung cancer (LC) and non-asbestos-related benign lung disease (BLD) patients in one study [74]. Two studies [70,71] mentioned MPM stages, most of which were Stage II or III (24 out of 33 subjects). Histopathologic subtypes were mentioned in two studies [70,74]. However, owing to the small sample sizes, none of the studies evaluated VOC changes in different subtypes or stages of MPM. The summary of individual articles is presented in Table 3 and Table 4.

**Figure 4:**
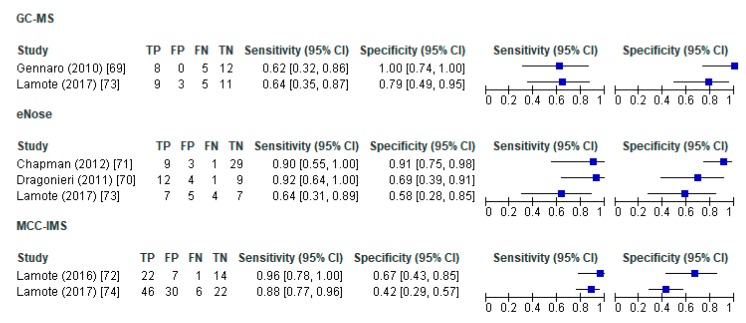
Forrest plots for sensitivity and specificity of VOC methods in distinguishing MPM from healthy controls. VOC—volatile organic compound; GC–MS—gas chromatography-mass spectrometer; MCC–IMS—multi-capillary column-ion mobility spectrometer; TP—true positives; FP—false positives; FN—false negatives; TN—true negatives; CI—confidence interval.

**Figure 5:**
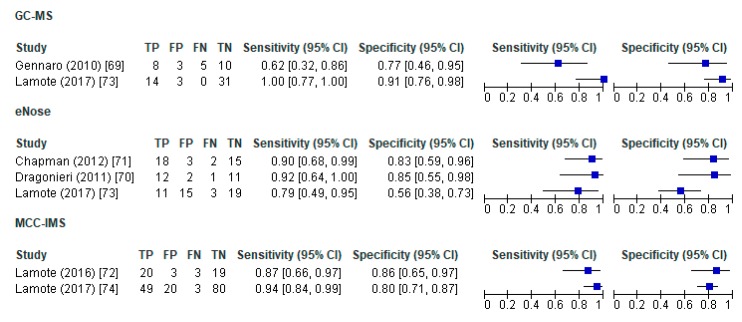
Forrest plots for sensitivity and specificity of VOC methods in distinguishing MPM from asbestos exposed. VOC—volatile organic compound; GC-MS—gas chromatography-mass spectrometer; MCC-IMS—multi-capillary column-ion mobility spectrometer; TP—true positives; FP—false positives; FN—false negatives; TN—true negatives; CI—confidence interval.

The forest plots for the sensitivity and specificity of VOC methods in distinguishing MPM from healthy controls showed that sensitivity (95% CI) changed between 0.62 (0.32–0.86) and 0.96 (0.78–1.0) across studies while specificity was between 0.42 (0.29–0.57) and 1.0 (0.74–1.0). Similarly, sensitivity to distinguish MPM from asbestos-exposed subjects was between 0.62 (0.32–0.86) and 0.96 (0.78–1.0) while specificity was 0.52 (0.32–0.71) and 1.0 (0.74–1.0). We observed heterogeneity across studies by comparing confound intervals visually. However, meta-analytic approaches (i.e., hierarchichal summary ROC, bivariate models, meta-regression) to pool diagnostic accuracy of studies and to address sources of heterogeneity, were not conducted due to the small sample sizes and the small number of studies under each VOC method (Figure 4 and Figure 5).

### 4.3. Exhaled Breath Collection and Analysis Methods

#### 4.3.1. Gas Chromatography Coupled to Mass Spectrometry (GC–MS) 

GC–MS was used as the analytical approach in two studies [69,72]. Gennaro et al. [69] tested the diagnostic accuracy of exhaled breath by using GC–MS in MPM diagnosis. Thirteen subjects with histologically proven MPM, 13 long-term asbestos-exposed subjects with radiologically proven benign pleural disease and 13 healthy subjects never exposed to asbestos were involved in the study. Breath samples were collected inside Tedlar^®^ bags after tidal breathing and a vital capacity maneuver. An inspiratory VOC-filter (A2, North Safety, Middelburg, The Netherlands) was used in order to reduce the contamination of ambient air. In addition, potential VOC emissions from the clean bags were accounted by monitoring background VOC concentrations inside clean Tedlar^®^ bags. The air inside Tedlar^®^ bags was pumped into sorbent cartridges composed of a cylindrical stainless-steel net with an external diameter of 4.8 mm, containing Carboxen 1003, Carbopack B and carbopack Y as adsorbent bad (Sigma Aldrich, Milano, Italy), subsequently. After thermal desorption of the cartridges by a thermal desorber (Markes International Ltd., Unity^TM^, Llantrisant, UK) equipped with an autosampler (Markes mod. ULTRA^TM^), the samples were analyzed by gas chromatography (Agilent GC-6890 PLUS) and a mass selective detector (Agilent MS-5973 N). The discriminant function analysis (DFA) shows that MPM patients showed higher concentrations of cyclohexane (median value = 251.79 ng/L) compared to subjects with long-term exposure to asbestos (median value = 69.31 ng/L) and healthy controls (median value = 33.08 ng/L), while cyclopentane was the dominant compound in the discrimination of asbestos exposed from MPM and healthy controls. They also showed the compounds that arise from the collector bags (i.e., DMCA, phenol). 

Six years later, Lamote et al. [72] recruited 14 treatment naïve MPM patients, 15 patients with benign asbestos-related diseases, 19 asbestos-exposed asymptomatic persons and 16 healthy controls in order to test the hypothesis that VOC patterns in exhaled breath would differ between MPM patients, asbestos-exposed and asbestos-unexposed subjects. They used GC–MS and e-nose for the breath analysis. Subjects breathed tidally into a two-way non-breathing valve (Hans Rudolph 2700, Hans Rudolph Kansas City, USA) during 5 min and exhaled full vital capacity volume into the 10L Tedlar^®^ bag, subsequently. An inspiratory VOC-filter (A2, North Safety, Middelburg, The Netherlands) was used to reduce the effect of exogenous VOCs. An amount of 500 ml of sample inside the bag was transferred to a sorbent tube (3.5 “long, 0.25 “outer diameter) filled with 200 mg Tenax^®^ GR (35/60 mesh; Merkes International Ltd., Llantrisant, UK), subsequently. Sorbent tubes were dry purged and breath samples were thermally desorbed using a Unity Series 2 Thermal Desorption system (Markes, Llantrisant, UK) coupled to GC–MS (thermo Finnigan, Austin, TX, USA). Diethyl ether, limonene, nonanal, methylcyclopentane and cyclohexane were found to be dominant VOCs to discriminate asymptomatic asbestos exposed from MPM, with an accuracy rate of 0.97. In addition, the GC–MS analysis was able to discriminate MPM patients from the pooled group of asymptomatic asbestos-exposed and benign asbestos disease patients with an accuracy of 0.94. 

#### 4.3.2. Ion Mobility Spectrometry Coupled to Multi-capillary Columns (IMS–MCC)

Breath Discovery ion mobility spectrometry (IMS) coupled to a multi-capillary column (MCC) method was used in two studies of the same research group [72,74]. In the first study [71], Lamote et al., investigated VOCs in exhaled breath of 23 MPM patients and compared the findings with those of 22 asymptomatic former asbestos exposed workers and 21 healthy asbestos non-exposed subjects. In both studies, breath samples were collected by a SpiroScout ultrasound-controlled breath sampler (Ganshorn Meizin Electronic, Niedelauer, Germany) connected to a BioScout breath analyzing device operating on VOCan v2.4 software (B&S Analytik, Dortmund, Germany). Alveolar air was sampled via monitoring CO_2_ levels in exhaled breath by the volumetric capnography method. Breath analytes initially passed within a non-polar OV-5 MCC column (Multichrom Ltd., Novobirsk, Russia), depending on their chemical properties. Pre-separated analytes were transferred to the ionization chamber where they became positively charged from prior-ionized carrier gas (α1-notrogen gas, Air Liquide Medical, 99.999% pure, CAS-no: 7727-37-9, Schelle, Belgium). Carrier gas was ionized by 95MBq 63 Ni β-radiation source and ionized molecules accelerated within an electrical field along the drift region. They eventually elicited an electrical current on a Faraday plate detector, leading to VOC peak intensity which was subsequently cross-checked with an IMS–MCC database. 

MPM patients were discriminated from both former asbestos-exposed and non-exposed healthy controls with an 87% sensitivity, a 70% specificity, and positive and negative predictive value of 61% and 91%, respectively. In addition, asbestos-exposed individuals could be discriminated from MPM patients with 87% sensitivity, 86% specificity, PPV and NPV of 87% and 86%, respectively. One year later, they aimed to validate the findings of the previous study in a larger population including subjects with different conditions. They were able to discriminate MPM from pooled group of asbestos exposed and benign asbestos-related diseases with a sensitivity of 95% and NPV of 96% [74]. They were also able to discriminate MPM from benign lung diseases and lung cancer with an accuracy of 80% and 72%, respectively. 

#### 4.3.3. Sensor-Based Technologies (Electronic Nose) 

An exhaled breath analysis was conducted by e-nose technology in three studies [70,71,73]. Dragonieri et al. [70] included 13 MPM, 13 subjects with previous long-term asbestos exposure history and 13 healthy control subjects and tested whether VOCs were able to discriminate MPM patients from asbestos-related benign pleural diseases and healthy controls. Patients breathed tidally into a non-breathing valve connected to an inspiratory VOC-filter (A2, North Safety, The Netherlands) and to a silica-filled drying chamber for 5 min. They subsequently performed a forced expiratory maneuver and exhaled into a Tedlar^®^ bag connected to Cyranose 320 device (Smiths Detections, Pasadena, CA, USA) containing a nanocomposite array with 32 sensors, each of which responds to exhaled breath by changing electrical resistance leading to unique breath prints. They found that MPM patients were discriminated from asbestos exposed subjects with a cross-validated accuracy (CVA) of 80.8%, a sensitivity of 92.3% and a specificity of 85.7%. Moreover, breath prints were able to differentiate MPM from healthy controls with a CVA of 84.6%. Chapman et al. [71] used carbon polymer assay (CPA) e-nose (Cyranose 320, Smiths Detections, Pasadena, CA, USA) to distinguish patients with MPM, asbestosis, benign asbestos-related pleural disease and healthy controls. They set up a two-phase study composed of a training and a validation phase conducted on different subjects. They collected breath samples into a 2-L gas impermeable bag (Rapak, Mulgrave, Australia), following tidal breathing and forced vital capacity maneuvers. However, a nose clip or VOC filter was not used during breath collection. The results showed that the breath prints of MPM patients were distinguished from those of control subjects with an accuracy of 95%. In addition, 38 out of 42 subjects were identified correctly in the validation phase. They concluded that for breath print identification between MPM, asbestos-related diseases, and healthy controls, CPA e-nose had a sensitivity of 90%, a specificity of 88%, a positive and negative predictive value of 60% and 97.8%, respectively. Lamote et al. [73] aimed to assess the accuracy of e-nose as well as that of GC–MS in MPM screening. Patients breathed tidally for 5 min and exhaled to their vital capacity into Tedlar^®^ bags. An amount of 500 mL of breath sample was adsorbed into a Tenax GR tube (Tenax GR SS 6 mm x 7” (CAMSCO, Houston, Texas, USA) and thermally desorbed, subsequently. Four different e-nose devices were used in the analysis (Cyranose C320, Tor Vergata eNose, Common Invent eNose, Owlstone Lonestar). Sensor signals introduced by four devices were combined to establish breath profiles. The e-nose technique was able to discriminate MPM from asbestos-exposed individuals (including asymptomatic and benign asbestos related disease patients) with 74% accuracy. In addition, they found sensitivity, specificity, positive and negative predictive values of e-nose in discriminating MPM from asbestos-exposed individuals of 0.82, 0.55, 0.82 and 0.55, respectively. 

### 4.4. Statistical Methods Used in Studies

#### 4.4.1. GC–MS Studies

Gennaro et al. [69] applied a variance analysis (ANOVA) on normalized data to find the compounds showing statistically significant difference in the exhaled breath of MPM, asbestos-exposed and healthy controls. A principal component analysis (PCA) and a discriminant function analysis (DFA) were used to find the greatest variance within data (PCA) and to find which variable discriminates between two or more groups (DFA). Statistica for Windows v. 6.1.144.0 (StatSoft Italia srl, Vigonza, Italy) was used for the analysis. In addition, counter propagation artificial neural networks (CP-ANN) were applied to data in order to obtain the best classification performance.

Lamote et al. [73] used the least absolute shrinkage and selection operator (lasso) regression analysis (glmnet R package v2.0-2) to find the VOCs that have the most discriminative power for distinguishing MPM from healthy controls as well as the number of times VOCs were selected. Model-predicted outcomes were used to construct an ROC curve (ROCR R-package- v1.0-7) and estimate sensitivity, specificity, positive and negative predictive values (PPV, NPV), the diagnostic accuracy of the final model, and the area under the receiver operating characteristics AUC(ROC). 

#### 4.4.2. IMS–MCC Studies

Raw data were processed by VisualNow v3.7 software (B&S Analytik, Dortmund, Germany), resulting in a list of VOC-peak intensities, in the two studies of Lamote et al. [72,74]. Due to the high number of variables and the low number of samples, they decided to use the lasso regression model (glmnet R-package). Model-predicted outcomes of all the patients were used to build a ROC curve (ROCR R-package- v1.0-7) and to estimate the sensitivity, specificity, positive and negative predictive values (PPV, NPV), and diagnostic accuracy of the final model and the AUC(ROC). In addition, the alveolar gradient of each VOC was calculated and added as a predictor in the model. 

#### 4.4.3. E-nose Studies 

In the study of Dragonieri et al. [70], raw data produced by sensors were analyzed by SPSS software version of 16.0 (SPSS Inc., Chicago, IL, USA). A t-test was used in order to select the principal components that are discriminative between the subject groups. Principal components were fit in a canonical discriminant analysis (CDA), subsequently to creating a model giving the maximum variance between groups and the minimum variance within the groups. The sensitivity, specificity, positive and negative predictive values as well as a ROC curve were developed on the basis of the canonical discriminant function. The cross-validated accuracy (CVA) percentage was calculated to find the agreement between the clinical and model-based classifications. 

Chapman et al. [71] also used principal component reduction and CDA and calculated CVA to find the accuracy of breath prints that were discriminative of different groups.

## 5. Discussion 

We identified six studies through our search criteria. The results regarding the accuracy of exhaled breath VOCs in discriminating MPM from healthy controls, as well as from asbestos-exposed individuals are encouraging. Gennaro et al., and Lamote et al. [69,73] reported that cyclohexane was able to discriminate MPM from the healthy subjects. Gennaro et al. [69] related this finding with the hypothesis of a relationship between reactions of xenobiotic agents’ degradation and the neoplastic processes [76,77] as cyclohexane is a metabolite of ε-caprolactam. Cyclohexane has also been proposed to be indicative of oxidative stress in the exhaled breath of lung cancer and colorectal cancer patients [78,79]. However, the exact mechanism underlying its endogenous origin is still not clear. Gennaro et al. [69] also found that cyclopentane was able to discriminate long-term asbestos-exposed subjects from MPM and healthy controls, concluding that it can be used as a screening marker in asbestos-exposed individuals. Similarly, Dragonieri et al. [70] and Chapman et al. [71] reported that e-nose was able to detect breathprints distinguishing MPM patients from asbestos exposed individuals. However, pattern-recognition techniques do not identify individual compounds but rather allow the recognition of the overall composition of VOCs. In addition, e-nose has a lower sensitivity compared to the gold standard GC–MS [73,80]. Lamote et al. [72,74] found a high sensitivity and NPV of VOC profiles (i.e., P3, P5, P50, P71) in discriminating MPM from asbestos-exposed subjects detected by IMS–MCC technique which supports the idea of using breath tests as a screening tool to discriminate MPM from former asbestos workers under the risk of developing MPM. However, the small sample size restricted researchers in identifying the possible molecular discriminators within the compounds and thus in interpreting the mechanisms underlying MPM pathogenesis. 

Although studies have shown promising results, we recognized some limitations regarding their qualities. First, all the studies were carried on small groups, which is likely to limit the internal validity of the methods as well as the generalizability of the findings to the population. All the studies involved histologically proven MPM cases which was in accordance with the global guidelines. However, there were heterogeneities among the control groups in terms of the selection of the subjects. For instance, cases and controls were not matched for age in some studies [69,70,73,74]. Nevertheless, it is difficult to find healthy, age-matched controls of MPM cases since most patients are diagnosed at old ages. Moreover, the effect of age on breath composition is not clear, as studies show conflicting results [71,81,82]. Similarly, the smoking status of cases and controls was altered among studies; for example, current smokers were excluded in two studies [70,71], cases and controls were matched for smoking status in one study [71], while in others, they were not excluded nor accounted for [72,73,74]. Lamote et al. [72] evaluated the predictive value of smoking and gender to discriminate MPM from asbestos-exposed and healthy controls by generalized linear model, yet smoking was not selected in the final model, indicating that it is not predictive of MPM even in asbestos-exposed population. Although smoking is not a cause of MPM, it has been well established that it may alter VOC composition in exhaled breath [82,83]. In addition to the exogenous compounds taken in by the inhalation of tobacco smoke, smoking may also induce oxidative stress-related changes leading to the production of endogenous VOCs. In light with those findings, we support the idea that VOC profiles should be further investigated in asbestos workers as their smoking rates are high [73]. 

In two studies [70,71], the control groups were selected from volunteers working in a hospital and in a university. Healthcare professionals are reported to be exposed to several VOCs, depending on occupation and work locations [84,85], although compelling evidence on exogenous VOCs from hospital environments contributing to the exhaled breath profiles of healthcare workers is hardly available. Despite the fact that there is no indication in the studies of de Gennaro et al. [70] and Dragonieri et al. [71] on exogenous exposure related with the hospital environment, we temperately recommend recruiting control groups that are not healthcare workers. 

Through our search strategy, we did not identify any studies regarding EBC in MPM patients. As such, EBC biomarkers were investigated in asbestos-exposed individuals in a limited number of studies. Chow et al. [46] reported elevated oxidative stress and inflammatory markers in asbestosis patients, but not in individuals with pleural diseases. Considering asbestosis increases the risk for developing MPM, we believe that EBC should be deeply investigated in MPM with regards to oxidative stress and inflammatory pathway mechanisms.

A limitation of our study is that we could not conduct a meta-analysis of the diagnostic accuracy as the small number of studies restricted us to address possible heterogeneity among studies using rigorous statistical methods.

## 6. Conclusions

MPM is the primary cancer of pleura associated with past asbestos exposure and recent reports predict an increase in incidence and mortality rates. Difficulties in diagnosing MPM at early stages lead to poor clinical outcome. Hence, significant efforts have been devoted to investigating serum and tissue biomarkers of MPM over the last two decades. As summarized in this review, research on exhaled breath biomarkers of MPM is at its early phases, yet is likely to be promising in the future, as breath is a non-invasive and repeatable way to access numerous biomarkers reflecting human metabolism. GC–MS and IMS–MCC are the analytical methods that have been used to assess VOC profiles in recent studies. Although GC–MS is the gold standard method, IMS–MCC has the advantage that no pre-concentration steps are needed; therefore, it can be utilized for a real-time breath analysis. The studies involved in this review identified some compounds (i.e, cyclohexane, P3, P5, P50, P71, diethyl ether, limonene, nonanal, VOC IK 1287) that can be indicative of MPM development in an asbestos-exposed population. Some of them (i.e., P3, P5, P50, P71) are needed to be studied further in terms of their composition in order to provide insights into their association with the MPM mechanism. In addition, VOC profiles should be interpreted carefully, as the respiratory system is the route to many exogenous substances and the elimination of endogenous VOCs may be affected by several individual factors (i.e., physiologic status, smoking, drugs etc.). We believe that exhaled breath studies need to account for the potential confounders that might have an impact on VOC composition. Pattern recognition techniques have also been used in some studies which showed promising results in discriminating MPM from asbestos-exposed and from healthy controls. Although the results on human studies are encouraging, small sample sizes and methodological diversities among studies limit their translation into clinical practice. Furthermore, a great majority of the studies lack external validation. Therefore, more prospective studies with standardized methodologies in line with the most recent guidelines should be conducted and external validation of the results should be tested on larger populations.

## Figures and Tables

**Table 1:** PECO worksheet.

Population	Malignant Pleural Mesothelioma Patients (MPM)
Exposure	Asbestos exposure
Comparison	MPM patients versus Asbestos exposed asymptomatic subjects (AEx) Subjects with benign asbestos related diseases (ARD) Healthy controls (HC)
Outcome	Exhaled breath profiles in MPM compared with that of (AEx, ARD, HC)

Note: MPM—malignant pleural mesothelioma; AEx—asbestos exposed; ARD—asbestos related benign diseases; HC—healthy controls.

**Table 2:** Quality assessment of the selected studies.

Study	Selection (/4)	Comparability (/2)	Exposure (/3)	Overall Score (/9)
Gennaro et al. (2010) [69]	3	1	1	5
Dragonieri et al. (2011) [70]	3	1	1	5
Chapman et al. (2012) [71]	4	1	1	6
Lamote et al. (2016) [72]	3	1	1	5
Lamote et al. (2017) [73]	3	1	1	5
Lamote et al. (2017) [74]	3	0	1	4

**Table 3:** Overview of the studies regarding study type and the exhaled breath sampling method.

Study	Study Type	Population (*n*, Age)	Breath Collection Method	Alveolar/Total Breath	Adjustment for Ambient Air and Collector
Gennaro (2010) [69]	Cross-sectional, case-control	MPM (13, 60.9 ± 12.2 y) AEx (13, 67.2 ± 9.8 y) HC (13, 52.2 ± 16.2 y)	Tidal breathing (5 min) followed by VC maneuver Tedlar bag	Total breath	Inspiratory VOC filter, Background VOC concentration in a clean Tedlar bag
Dragonieri (2011) [70]	Cross-sectional, case-control	MPM (13, 61 ± 12 y) AEx (13, 67 ± 10 y) HC (13, 52 ± 16 y)	Tidal breathing (5 min) followed by VC maneuver10 L Tedlar bag	Total breath	Inspiratory VOC filter, Background VOC concentration in a clean Tedlar bag
Chapman (2012) [71]	Multicenter, cross-sectional, case-control	MPM (20, 69 ± 10 y) Asbestosis (5, 70 ± 10.5 y) BLD (13, 70.9 ± 8.2 y) HC (42, 66.5 ± 14 y)	Tidal breathing (5 min) followed by VC maneuver 2 L gas impermeable bag	Total breath	-
Lamote (2016) [72]	Multicenter, cross-sectional, case-control	MPM (23, 66(59–73) y) AEx (22,56(55–57) y) HC (21,56(40–58) y)	Tidal breathing (3 min)	Alveolar breath	10 mL of ambient air sampled as background, alveolar gradients of VOCs were calculated
Lamote (2017) [73]	Multicenter, cross-sectional, case-control	MPM (14, 69(65–73) y) AEx (19,50(49–53) y) ARDs (15, 60(58–63) y) HC (16,56(52–59) y)	Tidal breathing (5 min) followed by VC maneuver 10 L Tedlar bag	Total breath	VOC filter Before sampling; Tenax tubes were being flushed with helium
Lamote (2017) [74]	Multicenter, cross-sectional, case-control	MPM (52, 67(62–72) y) AEx (59, 53(50–55) y) ARD (41, 58(55–62) y) BLD (70, 58(40–68) y) HC (52, 51(34–56) y)	Tidal breathing (3 min)	Alveolar breath	Disposable mouthpieces and filters Alveolar gradients of VOCs were calculated

Note: Mean [69,70,71], Median [72,73,74]. y—year; MPM—malignant pleural mesothelioma; AEx—asbestos exposed; HC—healthy controls; ARD—asbestos related benign diseases; BLD—non-asbestos related benign lung diseases—LC—lung cancer; VC—vital capacity VOC—volatile organic compounds.

**Table 4:** Overview of studies regarding breath detection and statistical methods used.

Study	Breath Profile Detection Method	Pre-Treatment	Statistics	Results
Gennaro (2010) [69]	GC–MS	Adsorbtion on thermal desorption (TD) sorbent cartridge	Shapiro-Wilk tests, ANOVA, PCA, DFA, CP-ANN	Cyclohexane able to discriminate MPM from HC and AEx Cyclopentane able to discriminate AEx from MPM and HC
Dragonieri (2011) [70]	E-nose	-	PCA, CDA	(1) MPM vs HC Accuracy = 0.84; Cut-off probability of MPM; diagnosis = 0.31; Sensitivity = 0.92; Specificity = 0.69; AUC(ROC) = 0.893 (2) MPM vs AEx Accuracy = 0.80; Cut-off probability of MPM; diagnosis = 0.33; Sensitivity = 0.92; Specificity = 0.85; AUC(ROC) = 0.91
Chapman (2012) [71]	E-nose	-	PCA, CDA, M-distance	(1) MPM vs HC Accuracy = 0.95; Sensitivity = 0.90; Specificity = 0.91 (2) MPM vs ARD Sensitivity = 0.90; Specificity = 0.83 (3) MPM vs ARD vs HC Sensitivity = 0.90; Specificity = 0.83
Lamote (2016) [72]	MCC–IMS	-	Chi-square/ Fisher’s exact, Kolmogorov-Smirnov, T-test/ANOVA, Wilcoxon-Mann-Whitney/ Kruskal-Wallis, Logistic regression using the least absolute shrinkage and selection operator (lasso)	(1) MPM vs HC Accuracy = 0.82; Sensitivity = 0.96; Specificity = 0.67; AUC(ROC) = 0.74; Selected VOCs = P50, P84 (2) MPM vs AEx Accuracy = 0.87; Sensitivity = 0.87; Specificity = 0.86, AUC(ROC) = 0.86; Selected VOCs = P3, P5, P30, P50, P54, P71 (3) AEx vs HC Accuracy = 0.91; Sensitivity = 0.95; Specificity = 0.86; AUC(ROC) = 0.94; Selected VOCs = P5, P8, P13, P25
Lamote (2017) [73]	GC–MS, E-nose	Adsorbtion onto Tenax GR sorbent tubes, Thermal desorption (TD)	Pearson’s Chi-square, Shapiro-Wilk, Lasso regression (applied to GC–MS data), PCA (applied to e-nose data)	GC–MS (1) MPM vs HC Accuracy = 0.71; Sensitivity = 0.64; Specificity = 0.79; AUC(ROC) = 0.77 (2) MPM vs AEx + ARD Accuracy = 0.94; Sensitivity = 1.0; Specificity = 0.91; AUC(ROC) = 0.94 (3) ARD vs AEx Accuracy = 0.5; Sensitivity = 0.60; Specificity = 0.42; AUC(ROC) = 0.36 Selected VOCs: diethyl ether, limonene, cyclohexane, nonanal, VOC IK 1287, isothiocyanatocyclohexane ENOSE (1) MPM vs HC Accuracy = 0.65; Sensitivity = 0.66; Specificity = 0.63; AUC(ROC) = 0.66 (2) MPM vs AEx + ARD Accuracy = 0.74; Sensitivity = 0.81; Specificity = 0.55; AUC(ROC) = 0.74 (3) ARD vs AEx Accuracy = 0.52; Sensitivity = 0.58; Specificity = 0.46; AUC(ROC) = 0.55
Lamote (2017) [74]	MCC–IMS	-	Fisher’s exact, Kolmogorov-Smirnov, ANOVA, Kruskal-Wallis	(1) MPM vs HC Accuracy = 0.65; Sensitivity = 0.89; Specificity = 0.43; AUC(ROC) = 0.61 (2) MPM vs AEx + ARD Accuracy = 0.85; Sensitivity = 0.94; Specificity = 0.80; AUC(ROC) = 0.89 (3) MPM vs LC Accuracy = 0.72; Sensitivity = 0.73; Specificity = 0.71; AUC(ROC) = 0.77 (4) MPM vs BLD Accuracy = 0.80; Sensitivity = 0.71; Specificity = 0.87; AUC(ROC) = 0.83

Note: GC–MS— Gas Chromatography Coupled to Mass Spectrometry; MPM—malignant pleural mesothelioma; AEx—asbestos exposed; HC—healthy controls; ARD—asbestos related benign diseases, BLD—non-asbestos related benign lung diseases; LC—lung cancer; VOC—volatile organic compounds; AUC—area under curve; ROC—receiver operating characteristics.

## Supplementary Materials

The following are available online at https://www.mdpi.com/1660-4601/17/3/1110/s1, Table S1: Search terms, Table S2: Quality assessment of selected studies.
