# Supplementary material for: Exhaled Breath Analysis in Diagnosis of Malignant Pleural Mesothelioma: Systematic Review
Source: Int J Environ Res Public Health. 2020 Feb 10;17(3):1110. doi: 10.3390/ijerph17031110 (PMC7036862; doi:10.3390/ijerph17031110)
Supplement: Supplementary file 1 [file ijerph-17-01110-s001.pdf]

**PUBMED**<br>**Concept: Mesothelioma**<br>"Mesothelioma"[Mesh] OR mesothelioma*[tiab] OR "Mesothelioma, Malignant" [Supplementary Concept] OR pleura mesothelioma*[tiab]<br>**Concept: Proteomics/biomarkers**<br>"Proteome"[Mesh] OR proteom*[tiab] OR<br>"Proteomics"[Mesh] OR<br>Phosphoproteom*[tiab] OR<br>"Breath Tests"[Mesh] OR "exhaled breath"[tiab] OR<br>Breath biops*[tiab]<br>Mass spectrometry[tiab] OR<br>Chromatography, Liquid[tiab] OR<br>2D MS[tiab] OR<br>SOMAMER[tiab]
**EMBASE**<br>**Concept: Mesothelioma**<br>'mesothelioma'/de OR mesothelioma*:ti,ab,kw OR<br>'pleura mesothelioma'/exp<br>**Concept: Proteomics/biomarkers**<br>'proteomics'/exp OR proteom*:ti,ab,kw OR<br>phosphoproteom*:ti,ab,kw OR<br>exhaled breath:ti,ab,kw OR<br>breath biops*:ti,ab,kw OR<br>'mass spectrometry:ti,ab,kw OR<br>Chromatography, Liquid:ti,ab,kw OR<br>2D MS:ti,ab,kw OR<br>'SOMAMER':ti,ab,kw OR<br>'exhaled breath condensate'/exp OR<br>'exhaled breath'/exp
**Web of Science**<br>TOPIC: (mesothelioma)<br>TOPIC: (proteom* OR Phosphoproteom* OR Proteogenomic* OR "exhaled breath condensate" OR "exhaled breath" OR "tumor marker" OR "tumor antigen" OR "mass spectrometry" OR "liquid chromatography" OR "2D LC MS" OR SOMAMER)

Table S1. Search terms

SELECTION			COMPARABILITY			EXPOSURE			SCORE
**Study**	**Adequate definition of case (*)**	**Representativeness of cases (*)**	**Selection of controls (*)**	**Definition of controls (*)**	**Comparability of cases and controls on the basis of the design or analysis<br>-Age, sex, smoking matching (*)<br>-Adjustment of additional factors (presence of other diseases) (*)**	**Ascertainment of exposure (maximum one (*)<br>-From company records, certificates (*)<br>-Structured interviews where investigator was blind to case/control status (*)**	**Same method of ascertainment for cases and controls (*)**	**Non-Response rate (*)**	**********<br>(9)**
**Gennaro et al (2010) (69)**	Yes, biopsy confirmed MPM *	Representative samples of MPM were recruited among patients visiting outpatient clinic of Occupational Medicine Department of University of Bari *	Group 1:Healthy controls were selected from volunteers working at hospital or university<br>Group 2: Asbestos related disease patients were recruited from Occupational Medicine Department of University of	No history of asbestos exposure or disease or drug use *	-Cases and controls were not matched for age or sex<br>-Any other respiratory, cardiovascular or systemic diseases, acute infections were excluded from both groups *	MPM: Self reported<br>ARD: Professional certificates*<br>Healthy controls: Self reported<br>No blinding of the investigator	No	No description of the response rate	*****<br>(5/9)

			Bari						
**Dragonieri et al (2011) (70)**	Yes, biopsy confirmed MPM *	Representative samples of MPM were recruited among patients visiting outpatient clinic of Occupational Medicine Department of University of Bari *	Group 1:Healthy controls were selected from volunteers working at hospital or university<br>Group 2: Asbestos related disease patients were recruited from Occupational Medicine Department of University of Bari	No history of asbestos exposure or disease or drug use *	-Cases and controls were not matched for age or sex<br>-Any other respiratory, cardiovascular or systemic diseases, acute infections were excluded from both groups *	MPM: Self reported<br>ARD: Professional certificates*<br>Healthy controls: Self reported<br>No blinding of the investigator	No	No description of the response rate	***** (5/9)
**Chapman et al (2012) (71)**	Yes,<br>Group 1: immunohistolo gical diagnosis of MPM<br>Group 2:history of professional asbestos exposure with an appropriate latency period and clinical and radiological signs of	Representative samples of MPM, asbestosis and pleural disease were recruited among patients visiting outpatient clinic of St Vincent's Hospital and Prince of Wales Hospital *	Volunteers from community *	No history of asbestos exposure or lung disease, no respiratory symptoms, normal spirometry*	-Cases and controls were matched for age, sex and smoking status *<br>-No information regarding any other coinciding disease	Self reported exposure status of all groups<br>No blinding of the investigator	Yes*	No description of the response rate	****** (6/9)

	asbestosis<br>Group 3: history of professional asbestos exposure with an appropriate latency period and clinical and radiological signs of pleural disease *								
**Lamote et al (2016) (72)**	Yes, treatment naïve MPM patients were recruited<br>MPM diagnosis was verified by Belgian Mesothelioma Pathology Panel *	Representative samples of MPM were recruited from University Hospitals of Ghent, Leuven and Antwerp *	Group 1: Healthy non-exposed controls were recruited from University Hospitals of Ghent, Leuven and Antwerpen<br>Group 2: Asymptomatic asbestos exposed controls (AEx) were recruited from a former fiber-cement factory	Group 1: Healthy non-exposed controls<br>Group 2: Asymptomatic asbestos exposed controls<br>Absence of asbestos related diseases of the controls were confirmed by recent CT-scan or chest x-ray < 12 months *	Gender and smoking status were adjusted with generalized linear model. *	AEx: From company records and questionnaires *<br>MPM: From questionnaires<br>No blinding of the investigator	No	No description of the response rate	***** (5/9)
**Lamote et al (2017) (73)**	Yes, treatment naïve MPM patients were recruited<br>MPM diagnosis	Representative samples of MPM were randomly recruited from	Healthy controls (group 1) and asbestos related lung diseases	Group 1: Healthy non-exposed controls<br>Group 2 :	-No matching or adjustion for age, sex, smoking status<br>-Any other non-	AEx: From company records and questionnaires *<br>MPM and HC:	No	No description of the response rate	**** (5/9)

	was verified by Belgian Mesothelioma Pathology Panel *	University Hospitals of Ghent, Leuven and Antwerp *	(ARD) (group 2) were recruited from University Hospitals of Ghent, Leuven and Antwerp Asymptomatic asbestos exposed were recruited from a former fiber-cement factory	Benign asbestos related Diseases Group 3: Asymptomatic asbestos exposed controls Medical conditions were confirmed by radiological investigations (recent CT and CXR) *	asbestos related diseases were excluded from controls *	From questionnaires No blinding of the investigator			
**Lamote et al (2017) (74)**	Yes, treatment naïve MPM patients were recruited MPM diagnosis was verified by Belgian Mesothelioma Pathology Panel *	Representative samples of MPM were randomly recruited from University Hospitals of Ghent, Leuven, Antwerp and OLV Hospital in Aalst *	Healthy controls (group 1), asymptomatic asbestos exposed controls (Aex) (group 2), asbestos related lung diseases (ARD) (group 3), primary lung cancer (LC) (group 4), lung diseases unrelated to asbestos exposure (BLD) (group 5) were randomly	Group 1: Healthy non-exposed controls Group 2: Asymptomatic asbestos exposed controls Group 3: Primary LC Group 4: Benign lung conditions unrelated to asbestos Group 5: Benign asbestos related diseases	-No matching or adjustion for age, sex, smoking status -No information regarding comorbid infection or systemic diseases within the groups	AEx and some of the ARD patients: From company records and questionnaires * MPM: From questionnaires No blinding of the investigator	No	No description of the response rate	**** (4/9)

			recruited from University Hospitals of Ghent, Leuven, Antwerp and OLV Hospital in Aalst. Some other asbestos related diseases and asymptomatic asbestos exposed (group 5) individuals were recruited from a former fiber-cement factory	Medical conditions were obtained via medical records and confirmed by radiological investigations (recent CT and CXR) *					

Table S2. Quality assessment of selected studies
